# Evaluation of Phytoavailability of Heavy Metals to Chinese Cabbage (*Brassica chinensis* L.) in Rural Soils

**DOI:** 10.1155/2014/309396

**Published:** 2014-09-11

**Authors:** Yao-Tsung Chang, Zeng-Yei Hseu, Franz Zehetner

**Affiliations:** ^1^Department of Environmental Science and Engineering, National Pingtung University of Science and Technology, Pingtung 91201, Taiwan; ^2^Institute of Soil Research, University of Natural Resources and Life Sciences, 1190 Vienna, Austria

## Abstract

This study compared the extractability of Cd, Cu, Ni, Pb, and Zn by 8 extraction protocols for 22 representative rural soils in Taiwan and correlated the extractable amounts of the metals with their uptake by Chinese cabbage for developing an empirical model to predict metal phytoavailability based on soil properties. Chemical agents in these protocols included dilute acids, neutral salts, and chelating agents, in addition to water and the Rhizon soil solution sampler. The highest concentrations of extractable metals were observed in the HCl extraction and the lowest in the Rhizon sampling method. The linear correlation coefficients between extractable metals in soil pools and metals in shoots were higher than those in roots. Correlations between extractable metal concentrations and soil properties were variable; soil pH, clay content, total metal content, and extractable metal concentration were considered together to simulate their combined effects on crop uptake by an empirical model. This combination improved the correlations to different extents for different extraction methods, particularly for Pb, for which the extractable amounts with any extraction protocol did not correlate with crop uptake by simple correlation analysis.

## 1. Introduction

Bioavailability of heavy metals in soils is critically dependent on the chemical speciation of the metals [1]. Plants respond only to the fraction that is “phytoavailable” to them. The readily soluble fraction of heavy metals is generally considered to be phytoavailable, but there is growing awareness that the current methods for assessment of “soluble” and “phytoavailable” fractions need reevaluation due to these fractions variability in both space and time [2]. The accurate estimation of heavy metal phytoavailability in soils is becoming more important as risk assessment and remediation efforts acknowledge that total metal concentrations may not be the best predictors of metal phytoavailability [3]. The most widely used methods for evaluating the phytoavailability of heavy metals in soils are single extraction and sequential extraction methods [4]. However, the sequential extraction methods are rather laborious and time consuming. Among single extraction methods, neutral salts, dilute acids, and chelating agents—all with only limited/varying success—have been the most widely used extractants besides resin-based techniques [5].

Water-soluble metal ions can be easily mobilized and may be considered as highly phytoavailable. To assess the readily bioavailable metal fractions under field conditions, collection and analysis of pore water have become an important aspect of environmental monitoring programs [4, 6, 7]. Understanding and modelling soil solution concentrations of heavy metals are indeed important in environmental assessment [8]. In this context, soil water extraction has the benefit of ascertaining metal concentrations at pseudoequilibrium in the soil solution. However, heavy metals can be adsorbed to negatively charged soil constituents, which are subject to cation exchange reactions, and can readily replenish soil solution levels. Weakly adsorbed metals are considered to be labile and available for plant uptake [3]. A wide variety of extractants have been proposed to ascertain metal exchangeability. Novozamsky et al. [9] proposed the use of 0.01 M CaCl_2_ as an extraction agent to estimate bioavailability of metals and nutrients in air-dried soil samples. However, Pueyo et al. [10] found systematically lower levels of extracted Cd, Cu, Pb, and Zn when using 0.1 M NaNO_3_ compared to 1 M NH_4_NO_3_ or 0.01 M CaCl_2_. In addition to single extractions, the application of sequential extraction, as proposed by Tessier et al. [11], provides estimates of the possible mobility and bioavailability of heavy metals in soil and sediment environments. Several sequential extraction methods have been employed to partition metals into fractions defined as soluble, exchangeable, organically bound, precipitated, oxide bound, and residual and to correlate metals in these fractions with plant concentrations or uptake [12–14]. Extraction with 0.11 M acetic acid (HOAc) is the first step of a three-step extraction procedure that was developed according to the Standards, Measurements, and Testing Program (formerly the European Community Bureau of Reference, generally termed the BCR method) [14]. The acidity of the HOAc induces dissolution of hydroxides and carbonates and increases overall metal solubility, whereas acetate induces additional metal mobilization by acting as a complexing agent. The use of HOAc can be of particular interest in bioavailability research because it is one of the most abundant low molecular weight organic acids (LMWOAs) present in the rhizosphere of many plants [15]. Baker and Amacher [16] defined the 0.1 M HCl-extractable fraction as the metals operationally released by moderate acid attack from soils. Additionally, complex extraction solutions, aimed at mimicking rhizosphere effects in the soil, have been developed to ascertain bioavailability of trace metals. Lindsay and Norvell [17] proposed a diethylenetriaminepentaacetic acid- (DTPA-) based extraction solution, buffered at pH 7.3 to exclude effects involving carbonate dissolution. This protocol is also widely used and predominant for phytoavailability studies. Also, ethylenediaminetetraacetic acid (EDTA) is often considered to provide a good estimate of the available soil metal concentrations [4, 18].

The soluble concentration of heavy metals in the soil solution is a poor indicator of phytoavailability because a portion of exchangeable as well as slightly complexed metals also contributes to the labile pool which can be easily taken up by roots [3, 4, 6, 18]. In addition, there is increasing evidence that plants can alter the chemical mobility and phytoavailability of heavy metals in the rhizosphere [19–21]. The rhizosphere pH is often more acidic than that of the bulk soil, and thus larger amounts of heavy metals are dissolved and possibly taken up in the vicinity of the root. Roots can also release soluble LMWOAs into the rhizosphere which are capable of complexing heavy metals and, hence, increase their potential uptake by plants [19]. With respect to these processes that are governed by the plants, bioassays conducted with popular crops certainly provide the best approach for evaluating heavy metal phytoavailability. Various indicators of the phytoavailability of the metals can be derived from such tests, with the most often used being the crop uptake of heavy metal, as estimated by analyzing the metal content of the crop [4, 6, 8, 22, 23]. The aims of this study were (1) to explore the extractability of heavy metals (Cd, Cu, Ni, Pb, and Zn) by different single extractions in rural soils varying in their degree of contamination, (2) to correlate the extractable concentrations of the metals with their uptake by a popular vegetable crop (Chinese cabbage), and (3) to develop an empirical model to predict the metal phytoavailability based on soil properties.

## 2. Materials and Methods

### 2.1. Soil Sample Collection

This study selected 22 surface soils (0–15 cm) to represent the major rural soils throughout Taiwan. These soils, derived from various parent materials, included a wide range of physical and chemical properties and degrees of heavy metal contamination. Entisols, Inceptisols, Andisols, Vertisols, Alfisols, Ultisols, and Oxisols (US soil classification system) were included in this study (Table 1). Their textures ranged from sandy loam to clay, and they differed in land use and agricultural production. Three of the soils contained elevated levels of Cd, Cu, Ni, Pb, or Zn due to illegal industrial wastewater discharge or hazardous solid waste disposal. Soil A from quaternary aged alluvium had been spiked with Cd and Pb salt for 1 yr prior to this study. Soil K derived from serpentine in eastern Taiwan contained high amounts of endogenous Cr and Ni from the parent material. Soil F was moderately contaminated by industrial wastewater. The remaining 16 soils were uncontaminated reference soils. All the soils were air-dried, ground, and passed through a 2 mm sieve for laboratory analyses.

### 2.2. Characterization of the Studied Soils

The pH was measured using a mixture of soil and deionized water (1 : 1, w/v) with a glass electrode [24]. Total organic carbon (OC) content was determined via the Walkley-Black wet oxidation method [25]. Cation exchange capacity (CEC) was determined with the ammonium acetate method (pH 7.0) [26]. Electrical conductivity (EC) was measured from the extract of a saturated paste of soil [27]. Total secondary free Fe (Fe_d_) was extracted with the dithionite-citrate-bicarbonate method [28]. An acid ammonium oxalate (pH 3.0) extraction was performed for noncrystalline (amorphous) Fe oxides [29]. Finally, soil particle-size distribution was determined with the pipette method [30]. Total contents of Cd, Cu, Ni, Pb, and Zn were measured after* aqua regia* digestion, following the procedure recommended by the International Organization for Standardization [31]. Samples were digested at room temperature with 37% HCl/70% HNO_3_ (3 : 1) mixture (28 mL per 3 g of sample) for 16 h. After this, the suspension was digested at 130°C for 2 h under reflux conditions. The suspension was then filtered and diluted to 100 mL with HNO_3_ 0.5 mol L^−1^ for analysis. Metal contents in all solutions were determined with a flame atomic absorption spectrophotometer (FAAS) (Hitachi Z-8100, Tokyo, Japan).

### 2.3. Single Extractions of Soil Heavy Metals

Six single extractions were undertaken to predict the labile pools of heavy metals in the soils (Table 2). The chemical extractants were distilled water, NaNO_3_, CaCl_2_, HOAc, HCl, EDTA, and DTPA. All extracts were centrifuged and further filtered by a Whatman No. 42 filter paper and <0.45 *μ*m Millipore filter paper. The metal concentrations in all the above solutions were determined by FAAS or ICP-OES (Optima 2100DV Model, Perkin-Elmer, USA). All soil samples were extracted and analyzed in triplicate. Additionally, Rhizon soil solution sampling was used (further described in the next section).

### 2.4. Pot Experiment of Chinese Cabbage

Air-dried soil (2 kg) was weighed and transferred into a plastic pot 10 cm in diameter. Chinese cabbage (*Brassica chinensis* L.) is a popular foliar crop in Taiwan. Seeds of Chinese cabbage were incubated at 25°C on filter papers in Petri dishes containing 10 mL distilled water for 5 days. Five germinated seeds were planted at a depth of 0.5 cm in the soil in each pot, which was fertilized with 50 mg N kg^−1^ ammonium sulfate, 50 mg P kg^−1^ calcium phosphate, and 40 mg K kg^−1^ potassium chloride, in a greenhouse. Greenhouse relative humidity was 70–90%, air temperature was 23–27°C, and day length was approximately 12 h. After 1 week, the seedlings were thinned to 1 per pot. The soil moisture content was adjusted daily to 75% of its water holding capacity by weighing the pots and adding deionized water to compensate for weight loss. The pot bottoms were sealed to eliminate leaching of mobilized heavy metals. This experiment was performed for all 22 soils in triplicate in a random block design; thus, 66 pots were used. The plant shoots, cut at the soil surface, were harvested 40 days after the seedling thinning. The soil was then broken up and roots were harvested by hand. The roots were washed in tap water until free of soil particles. The shoots and roots were further washed with deionized water, oven-dried at 70°C for 24 h, weighed, and then ground and passed through a 1.0 mm sieve. Aliquots of plant powder (0.5 g dried weight) were digested overnight in 14 M HNO_3_ (5 mL) and 30% H_2_O_2_ v/v (10 mL) and heated at 120°C for 2 h [32]. The digested solutions were filtered using Whatman No. 42 filter paper and diluted to 50 mL with deionized water. The concentrations of heavy metals in the digested solution were determined by ICP-OES. Additionally, the Rhizon soil solution sampler (2 mm in diameter) with no ion exchange capacity (RSMS: rhizosphere research product, Eijkelkamp, Giesbeek, The Netherlands) was inserted vertically in the center of each pot. Soil solution samples were collected by suction using a syringe at plant harvest (day 40). The metals in these solutions were directly analyzed by ICP-OES.

### 2.5. Quality Assurance, Quality Control, and Statistical Analysis

Two standard reference materials, BCR-141R (loam soil) and SRM 1573a (tomato leaves), were digested in triplicate and analyzed using* aqua regia* and the plant digestion method described. A good agreement was observed between the measured and the certified values for the metals analyzed (Cd, Cu, Ni, Pb, and Zn), with recovery ranging from 85 to 110%. A blank was run for each extraction procedure to correct the measurements. For sets of every ten samples, a procedure blank and spiked sample, involving all reagents, was performed to check for interference and cross contamination. The limit of detection, calculated as 3 *s*/*m* (where *s* is the standard deviation of the blank and *m* is the slope of the calibration curve), for each element determined was (mg L^−1^) Cd, 0.005; Cu, 0.01; Ni, 0.02; Pb, 0.04; and Zn, 0.001 for the FAAS measurements. However, the limit of detection for each element determined was (*μ*g L^−1^) Cd, 0.1; Cu, 0.6; Ni, 0.01; Pb, 2.0; and Zn, 0.1 for the ICP-OES measurements. Descriptive statistics, Pearson correlations, and significance analysis (*P* = 0.01; 0.05) were performed using SPSS 11.0 (SPSS Inc.) and Excel (Microsoft Inc.) software packages.

## 3. Results and Discussion

### 3.1. Soil Characteristics and Total Metals

The soils used for this study varied widely in their properties (Table 3). Soil pH values ranged from 4.1 to 8.8, while soils A, B, C, D, and E were strongly acidic, soils Q, R, S, T, U, and V were strongly alkaline, and the others tended to be neutral. All soils were characterized by low and medium organic carbon contents (ranging from 0.3 to 4.3%), typical for humid tropical regions, except for soil C, which were derived from volcanic ash. Because of the wide range of soil texture, the CEC values varied from 3.5 to 50 cmol kg^−1^. The soils had medium to high clay contents ranging from 10 to 47%. Comparing to the soil control standards (SCS) for rural lands in Taiwan, it became obvious that heavy contamination of Cd, Cu, Ni, Pb, and Zn occurred in several soils including soils A, H, K, T, and U (Table 4).

### 3.2. Comparison of Extractable Metals Using Various Extraction Methods

Table 5 lists the ranges and means of metal concentrations obtained with different extraction methods. The metal concentrations collected by the 1 : 5 soil-water extract from the air-dried soils were higher than those by the Rhizon method with suction from the field-capacity soils. However, the amounts of metals brought into solution by water from a soil are usually very low, and thus the metal concentrations with water extraction and the Rhizon method were much lower than those with the other methods (Table 5). Of all the single extractions in this study, HCl was theoretically the most aggressive agent for removing metals from the soil, and thus HCl-extractable metals were the highest. Generally, the extractable metals followed the descending order HCl > EDTA > DTPA > HOAc > CaCl_2_ > NaNO_3_ > H_2_O > Rhizon method. In addition to HCl, chelating agents EDTA and DTPA as well as HOAc extracted more metals from the soils than CaCl_2_ and NaNO_3_. The reasons for this extraction order are easily understood. EDTA is a strong and nonspecific chelating agent; it can extract labile and nonlabile fractions of metals in soil and has been reported to remove both organically bound metals and metals occluded in oxides and secondary clay minerals in part [13]. However, DTPA was originally developed for near-neutral and calcareous soils as it is buffered at pH 7.3 and therefore minimizes dissolution of carbonates [17], although uncertainty concerning the dissolution of iron and aluminum compounds remains [13].

The 0.11 M HOAc was used in the first step of the above-mentioned BCR method. Acetic acid can extract organic matter-bound metals in part and release most of the metals associated with carbonates and minerals such as kaolinite and ferrihydrite [33]. On the other hand, with the soil background electrolyte solutions CaCl_2_ and NaNO_3_, easily exchangeable metals can be extracted [10]. However, lower concentrations of metals were generally extracted with NaNO_3_ than with CaCl_2_ because monovalent cations exert only weak competition for adsorption sites on organic matter [9].

### 3.3. Simple Correlation Analysis between Single Extractions and Plant Concentrations

The correlations between extractable metals from soil pools and plant metal concentrations were better for shoots than roots (Table 6). However, for Cd and Pb, the correlations were low and mostly nonsignificant. For any of the studied metals, the concentrations in roots or shoots of Chinese cabbage were not significantly (*P* < 0.05) correlated with those in the Rhizon soil solution sampler, even though the Rhizon samplers were originally designed for seepage water sampling in the unsaturated zone [4]. This result suggests that the plants took up heavy metals from pools other than seepage water. The edible part of Chinese cabbage is the shoot, and the correlation coefficients between extractable metals in soil pools and metals in shoots were higher than in roots. Thus, the following discussion was focused on the relationship between Cu, Ni, and Zn in shoots and in soil pools. The concentrations of Cu, Ni, and Zn in the shoots of Chinese cabbage were significantly and positively correlated with the metals extracted by water, NaNO_3_, CaCl_2_, HOAc, HCl, EDTA, DTPA, and* aqua regia*, suggesting that these extraction procedures provided good measures for Cu, Ni, and Zn phytoavailability under the current experimental conditions. However, the correlations were always better for Cu and Ni compared to Zn.

### 3.4. Soil Property Considerations of Single Extractions

Table 7 demonstrates the relationships between the metal concentration in the different extractable fractions and selected soil properties including pH, OC, CEC, EC, Fe_o_/Fe_d_, and clay content. These soil properties have previously been found to control the solubility and activity of heavy metals in soils and to affect metal phytoavailability [3]. The chemical extraction methods are based on the assumption that there is a relationship between the extractable fraction of metals and the phytoavailability of the metals to plants; a good correlation is therefore supposed to reflect that the particular soil metal fraction is available to plants. However, a statistically significant correlation of the soil-to-plant system does not necessarily provide insight into the mechanisms of phytoavailability [20]. Different physical, chemical, and biological mechanisms may govern the translocation of different metals from soils to plants [3]. We observed highly variable correlations between extractable metal concentrations and soil properties (Table 7), which may reflect the different above-mentioned extraction mechanisms. Overall, pH and clay content were the most important soil properties affecting metal extractability, which is consistent with previous findings [3].

Considering this situation, a stepwise multiple linear regression analysis was performed. Soil pH, clay content, total metal contents (by* aqua regia*) of soils, and extractable metal concentrations were considered to simulate their combined effects on plant metal uptake. An empirical model was derived to express the relationships between metal phytoavailability and soil properties as follows:

(1) Metalshoot=a+bMetalaqua  regia +cMetalextraction+dpH+eClay,

where Metal_shoot_ and Metal_aqua  regia_ are the total metal concentrations in plant shoots and in soils, respectively. Metal_extraction_ is the metal concentration in the extractable soil fractions. The data of Cd and Pb in soil A were excluded for running this model because highly spiked Cd and Pb levels caused a clear bias of the normality *t*-test. Additionally, Chinese cabbage could not be harvested in the soils T, U, and V because their pH was too high to cultivate the crop. Therefore, the *n* value for Cd and Pb was 17 and that for Cu, Ni, and Zn is 18 for this model. For simplicity, the coefficients of the empirical equations are omitted. The coefficient of determination, *r* ^2^, allows an evaluation of the overall prediction of phytoavailability (Table 8). In general, the inclusion of soil property variables improved the correlations to different extents for different extraction methods, particularly for Pb, for which the extractable amounts were not correlated with plant uptake by simple correlation analysis. Nevertheless, this model still performed poorly in predicting plant uptake of Cd.

## 4. Summary and Conclusions

The metal levels extracted with all the single extraction protocols employed in this study were much higher than those with the Rhizon soil solution sampler. The extractable metals followed the descending order HCl > EDTA > DTPA > HOAc > CaCl_2_ > NaNO_3_ > H_2_O > Rhizon method. Low correlation was observed between extractable Cd and Pb in the soils and their respective contents in the roots and shoots of Chinese cabbage. Because of the different extraction mechanisms utilized by the different methods, correlations between extractable heavy metals and soil properties were highly variable. However, pH and clay content were identified as the most important soil properties affecting metal extractability. Soil pH, clay content, total metal contents of soils, and extractable metal concentrations were considered together in an empirical model to simulate their combined effects on plant uptake. The inclusion of soil pH and clay content improved the correlations with plant uptake to different extents for different extraction methods, particularly for Pb, for which the extractable amounts by any of the tested extraction methods were not correlated with plant uptake by simple correlation analysis. Our study shows that soil properties should additionally be considered when predicting heavy metal phytoavailability from soil extractions.

## Figures and Tables

**Table 1:** Description of the studied soils.

Soil code	Classification	Texture	Parent materials	Land use	Contamination with heavy metal
A	Typic Hapludox	Clay	Quaternary aged alluvium	Tropical orchard	Spiked Cd and Pb for 1 yr
B	Typic Paleudult	Sandy clay loam	Quaternary aged alluvium	Grass	Low
C	Typic Melanudand	Silty clay loam	Volcanic ash	Secondary tropical forest	Low
D	Typic Hapludult	Clay loam	Quaternary aged alluvium	Sugarcane	Low
E	Typic Udorthent	Sandy clay loam	Andesite and limestone	Grass	Low
F	Typic Fluvaquent	Silty clay loam	Slate alluvium	Lowland rice	Moderate
G	Typic Paleudalf	Silty clay loam	Slate alluvium	Soybean	Low
H	Typic Eutrudept	Silty clay loam	Slate alluvium	Lowland rice	High
I	Typic Paleudalf	Clay loam	Sandstone and shale alluvium	Sugarcane	Low
J	Typic Paleudalf	Loam	Coral reef and sandstone mixture	Virgin tropical forest	Low
K	Typic Hapludert	Clay loam	Serpentine	Secondary tropical forest	Endogenous Cr and Ni
L	Typic Eutrudept	Silty clay loam	Slate alluvium	Vineyard	Low
M	Typic Udorthent	Loam	Slate alluvium	Corn	Low
N	Lithic Udipsamment	Loam	Slate alluvium	Sugarcane	Low
O	Typic Eutrudept	Silty clay loam	Slate and sandstone alluvium	Tropical orchard	Low
P	Typic Udorthents	Sandy clay loam	Slate alluvium	Melon	Low
Q	Lithic Udorthent	Sandy loam	Schist alluvium	Sugarcane	Low
R	Typic Udorthents	Sandy loam	Sandstone and shale alluvium	Vegetable	Low
S	Typic Hapludalf	Clay loam	Coral reef	Secondary tropical forest	Low
T	Lithic Udipsamment	Sandy loam	Slate alluvium	Fallow	High
U	Typic Urothent	Silt loam	Slate alluvium	Fallow	High
V	Typic Urothent	Clay loam	Mudstone	Soybean	Low

**Table 2:** Single extraction procedures used.

Extraction method	Liquid : solid ratio	Equilibration time	Reference
Rhizon soil solution samplers	At field capacity	40-day incubation	
Distilled water	5 : 1	16 h	Mench et al. [34]
0.1 M NaNO_3_	5 : 2	2 h	Gupta and Aten [35]
0.01 M CaCl_2_	10 : 1	3 h	Novozamsky et al. [9]
0.11 M HOAc	20 : 1	16 h	Ure et al. [36]
0.1 N HCl	10 : 1	1 h	Baker and Amacher [16]
0.05 M EDTA	10 : 1	1 h	Wear and Evans [37]
0.005 M DTPA, 0.01 M CaCl_2_, and 0.1 M TEA	2 : 1	2 h	Lindsay and Norvell [17]

**Table 3:** Selected properties of the studied soils.

Soil code	pH	OC^a^	CEC^b^	EC^c^	Fe_o_^d^	Fe_d_^e^	Clay
		%	Cmol kg^−1^	dS m^−1^	g kg^−1^		%
A	4.1	1.3	3.9	0.20	4.47	19.4	47
B	4.5	0.8	10	0.19	0.50	6.7	28
C	4.5	17	50	0.31	2.51	18.6	34
D	4.8	2.1	9.9	0.26	3.66	7.11	33
E	5.3	4.2	3.5	0.21	1.40	1.54	18
F	5.6	3.1	5.0	0.82	6.95	8.37	37
G	5.6	4.3	13	1.30	2.44	8.49	27
H	6.1	3.8	17	0.51	8.62	9.29	38
I	6.1	1.5	9.9	0.66	2.89	6.60	29
J	6.4	1.9	7.8	2.58	1.08	14.4	25
K	6.5	2.9	32	0.32	0.83	14.0	36
L	7.0	1.4	9.7	2.54	2.35	5.43	31
M	7.0	1.5	7.7	1.08	1.62	6.27	24
N	7.1	0.8	8.5	1.40	2.21	7.07	19
O	7.3	1.6	8.3	1.06	2.70	9.23	30
P	7.5	0.3	8.9	1.42	0.97	14.0	18
Q	7.6	2.3	4.4	0.87	1.40	2.25	10
R	7.7	1.3	5.9	0.37	1.68	6.27	18
S	7.8	1.6	9.6	0.42	0.75	6.80	29
T	8.1	1.1	4.4	0.53	3.37	3.98	15
U	8.2	1.6	3.9	16.5	2.19	6.53	20
V	8.8	0.6	8.5	10.7	2.26	1.98	30

^a^Organic carbon.

^b^Cation exchange capacity.

^c^Electrical conductivity.

^d^Oxalate extractable iron.

^e^Dithionite-citrate-bicarbonate extractable iron.

**Table 4:** Total contents of heavy metals in the studied soils and the soil control standards (SCS) for rural lands in Taiwan.

Soil code	Cd	Cu	Ni	Pb	Zn
	mg kg^−1^				
A	18.9	18.4	21.0	965	47.8
B	0.55	10.1	35.3	41.5	16.8
C	1.05	57.6	12.3	41.5	66.2
D	0.71	23.9	24.4	29.6	69.6
E	0.87	17.5	26.0	3.74	34.9
F	0.91	16.4	53.1	25.8	39.4
G	0.74	30.5	44.1	25.8	67.8
H	1.57	475	407	36.6	607
I	0.74	12.1	19.7	17.1	72.4
J	0.90	14.0	31.4	15.1	44.9
K	0.46	46.8	2210	13.5	85.0
L	1.25	38.3	30.2	21.6	153
M	1.05	23.9	33.3	20.5	104
N	0.45	22.5	17.6	17.4	97.8
O	1.10	48.7	37.8	35.0	187
P	0.33	15.4	24.7	10.0	67.7
Q	1.34	18.7	18.5	15.1	39.1
R	1.02	9.60	20.8	14.4	52.8
S	1.53	20.0	26.3	36.8	142
T	3.60	1820	1170	2690	2740
U	6.25	2270	878	2100	998
V	1.02	18.8	37.4	22.1	84.3

SCS	5.00	200	200	500	600

**Table 5:** Ranges and means of extractable heavy metals in the studied soils. The unit for the Rhizon soil solution sampler is *μ*g L^−1^, and the others are mg kg^−1^.

Element	Rhizon		H_2_O		NaNO_3_		CaCl_2_		HOAc		HCl		EDTA		DTPA	
	Range	Mean	Range	Mean	Range	Mean	Range	Mean	Range	Mean	Range	Mean	Range	Mean	Range	Mean
Cd	0.24–128	8.33	0.06–0.38	0.10	0.06–7.18	0.40	0.02–14.5	0.70	0.04–12.1	0.92	0.11–19.5	1.30	0.15–18.9	1.29	0.06–16.4	0.90
Cu	0.77–105	16.7	0.28–1.55	0.49	0.14–0.77	0.31	0.02–1.16	0.12	0.06–11.0	1.18	0.37–1630	95.4	1.04–1090	107	0.39–332	36.9
Ni	0.01–1670	115	0.20–10.3	0.96	0.10–2.91	0.36	0.05–9.53	0.74	0.20–125	9.90	0.60–330	42.2	0.17–88.2	11.5	0.11–38.1	3.69
Pb	2.00–33.6	8.89	0.01–6.64	0.35	0.18–15.5	1.09	0.19–41.9	2.35	0.04–33.0	1.77	3.00–1850	187	1.05–1750	169	0.44–429	50.0
Zn	0.20–4310	225	0.03–1.26	0.14	0.04–4.75	0.74	0.06–15.9	1.52	0.03–4.06	0.77	0.86–1400	94.4	0.55–638	63.5	0.47–119	16.0

**Table 6:** Pearson linear correlation coefficients between heavy metal extracted from the soil with different extraction methods and concentrations in the root and shoot of Chinese cabbage (*n* = 17 for Cd and Pb; *n* = 18 for Cu, Ni, and Zn).

	Cd	Cu	Ni	Pb	Zn
Root					
Rhizon	−0.19	−0.25	−0.05	0.05	−0.12
H_2_O	−0.21	0.69∗∗	0.97∗∗	−0.30	0.50
NaNO_3_	0.04	0.69∗∗	0.27	−0.14	0.50
CaCl_2_	−0.28	0.79∗∗	0.19	−0.21	0.53∗
HOAc	−0.09	0.71∗∗	0.21	0.55∗	0.43
HCl	−0.30	0.68∗∗	0.56∗	0.20	0.51
EDTA	−0.28	0.66∗∗	0.80∗∗	−0.33	0.52∗
DTPA	−0.24	0.67∗∗	0.64∗∗	−0.20	0.54∗
*Aqua regia *	−0.21	0.69∗∗	0.97∗∗	−0.18	0.45

Shoot					
Rhizon	−0.05	−0.13	−0.07	−0.14	−0.06
H_2_O	0.06	0.89∗∗	0.72∗∗	−0.06	0.65∗∗
NaNO_3_	0.31	0.69∗∗	0.80∗∗	−0.37	0.74∗∗
CaCl_2_	0.14	0.93∗∗	0.75∗∗	−0.40	0.73∗∗
HOAc	0.04	0.90∗∗	0.75∗∗	0.27	0.75∗∗
HCl	−0.22	0.91∗∗	0.94∗∗	0.17	0.66∗∗
EDTA	−0.14	0.91∗∗	0.97∗∗	0.21	0.66∗∗
DTPA	−0.04	0.91∗∗	0.96∗∗	−0.13	0.69∗∗
*Aqua regia *	0.11	0.91∗∗	0.80∗∗	0.12	0.60∗∗

**Significant at the 0.01 level, *significant at the 0.05 level.

**Table 7:** Pearson linear correlation coefficients between extractable heavy metal concentrations and soil properties (*n* = 22).

	pH						OC				
Extraction	Cd	Cu	Ni	Pb	Zn		Cd	Cu	Ni	Pb	Zn
Rhizon	−0.43∗	0.11	−0.08	−0.30	−0.09		−0.08	−0.07	−0.07	−0.11	−0.07
H_2_O	−0.24	0.21	−0.01	−0.41∗	−0.19		−0.20	−0.02	0.05	−0.08	0.01
NaNO_3_	−0.41∗	−0.35	−0.11	−0.45∗	−0.27		−0.08	0.53∗∗	0.12	−0.06	0.34
CaCl_2_	−0.41∗	−0.26	−0.06	−0.42∗	−0.14		−0.08	0.18	0.06	−0.07	0.16
HOAc	−0.37	−0.20	−0.32	−0.41∗	−0.38		−0.11	0.13	0.93∗∗	−0.08	0.31
HCl	−0.36	0.25	0.34	0.23	0.27		−0.09	−0.08	−0.07	−0.14	−0.09
EDTA	−0.35	0.37	0.13	0.27	0.36		−0.09	−0.09	0.01	−0.13	−0.10
DTPA	−0.39	0.35	−0.04	−0.12	0.24		−0.09	−0.08	0.07	−0.14	−0.03
*Aqua regia *	−0.26	0.38	0.20	0.27	0.36		−0.10	−0.09	−0.04	−0.13	−0.10

	CEC						EC				
Extraction	Cd	Cu	Ni	Pb	Zn		Cd	Cu	Ni	Pb	Zn

Rhizon	−0.16	−0.10	−0.03	−0.10	−0.03		−0.12	0.15	−0.07	−0.14	−0.09
H_2_O	−0.22	0.01	0.47∗	−0.15	0.04		−0.02	0.27	−0.12	−0.11	−0.16
NaNO_3_	−0.15	0.41	0.27	−0.13	0.36		−0.10	0.20	−0.12	−0.12	0.00
CaCl_2_	−0.15	0.21	0.19	−0.14	0.18		−0.10	−0.13	−0.09	−0.11	−0.02
HOAc	−0.17	0.14	0.86∗∗	−0.14	0.30		−0.07	−0.12	−0.13	−0.10	−0.24
HCl	−0.17	−0.11	−0.04	−0.25	−0.12		−0.07	−0.10	0.50	0.37	−0.04
EDTA	−0.17	−0.17	0.32	−0.23	−0.16		−0.02	0.43	0.01	0.39	0.34
DTPA	−0.16	−0.15	0.37	−0.24	−0.07		−0.07	0.55	−0.11	0.00	0.36
*Aqua regia *	−0.21	−0.17	0.29	−0.24	−0.16		0.13	0.59	0.15	0.41∗	0.34

	Fe_o_/Fe_d_						Clay				
Extraction	Cd	Cu	Ni	Pb	Zn		Cd	Cu	Ni	Pb	Zn

Rhizon	−0.12	0.00	−0.07	−0.38	0.03		0.52∗∗	−0.17	0.03	0.37	0.07
H_2_O	−0.11	0.32	−0.20	−0.12	0.36		0.44∗	0.05	0.21	0.52∗∗	0.37
NaNO_3_	−0.13	0.05	0.25	−0.14	0.22		0.50∗∗	0.20	0.32	0.51∗∗	0.38
CaCl_2_	−0.13	0.29	0.29	−0.14	0.33		0.51∗∗	0.42∗	0.30	0.51∗∗	0.32
HOAc	−0.18	0.37	−0.10	−0.12	0.30		0.48∗∗	0.31	0.26	0.51∗∗	0.46∗
HCl	−0.09	0.37	0.22	0.16	0.36		0.47∗	−0.24	−0.16	−0.18	−0.25
EDTA	−0.10	0.28	0.14	0.18	0.32		0.46∗	−0.29	0.16	−0.22	−0.27
DTPA	−0.12	0.27	0.13	0.07	0.37		0.50∗∗	−0.22	0.35	0.19	−0.05
*Aqua regia *	−0.07	0.20	−0.04	0.16	0.32		0.40	−0.29	0.04	−0.21	−0.27

**Significant at the 0.01 level, *significant at the 0.05 level.

**Table 8:** Coefficients of determination (*r* ^2^) obtained by multiple linear regression (*n* = 17 for Cd and Pb; *n* = 18 for Cu, Ni, and Zn).

	Rhizon	H_2_O	NaNO_3_	CaCl_2_	HOAc	HCl	EDTA	DTPA
Cd	0.29	0.40	0.37	0.27	0.27	0.30	0.28	0.27
Cu	0.86∗∗	0.86∗∗	0.86∗∗	0.89∗∗	0.88∗∗	0.90∗∗	0.89∗∗	0.90∗∗
Ni	0.72∗∗	0.95∗∗	0.95∗∗	0.95∗∗	0.95∗∗	0.96∗∗	0.96∗∗	0.96∗∗
Pb	0.58∗∗	0.50∗	0.52∗	0.53∗	0.49∗	0.49∗	0.49∗	0.53∗
Zn	0.43	0.45∗	0.61∗∗	0.67∗∗	0.58∗∗	0.56∗	0.54∗	0.62∗∗

**Significant at the 0.01 level, *significant at the 0.05 level.
